# Electroencephalographic Brain Dynamics Following Manually Responded Visual Targets

**DOI:** 10.1371/journal.pbio.0020176

**Published:** 2004-06-15

**Authors:** Scott Makeig, Arnaud Delorme, Marissa Westerfield, Tzyy-Ping Jung, Jeanne Townsend, Eric Courchesne, Terrence J Sejnowski

**Affiliations:** **1**Swartz Center for Computational Neuroscience, Institute for Neural ComputationUniversity of California at San Diego, La Jolla, California, United States of America; **2**Department of Neurosciences, University of California at San DiegoLa Jolla, California, United States of America; **3**Children's Hospital Research Center, San DiegoCalifornia, United States of America; **4**Howard Hughes Medical Institute, Computational Neurobiology LaboratorySalk Institute for Biological Studies, La Jolla, CaliforniaUnited States of America

## Abstract

Scalp-recorded electroencephalographic (EEG) signals produced by partial synchronization of cortical field activity mix locally synchronous electrical activities of many cortical areas. Analysis of event-related EEG signals typically assumes that poststimulus potentials emerge out of a flat baseline. Signals associated with a particular type of cognitive event are then assessed by averaging data from each scalp channel across trials, producing averaged event-related potentials (ERPs). ERP averaging, however, filters out much of the information about cortical dynamics available in the unaveraged data trials. Here, we studied the dynamics of cortical electrical activity while subjects detected and manually responded to visual targets, viewing signals retained in ERP averages not as responses of an otherwise silent system but as resulting from event-related alterations in ongoing EEG processes. We applied infomax independent component analysis to parse the dynamics of the unaveraged 31-channel EEG signals into maximally independent processes, then clustered the resulting processes across subjects by similarities in their scalp maps and activity power spectra, identifying nine classes of EEG processes with distinct spatial distributions and event-related dynamics. Coupled two-cycle postmotor theta bursts followed button presses in frontal midline and somatomotor clusters, while the broad postmotor “P300” positivity summed distinct contributions from several classes of frontal, parietal, and occipital processes. The observed event-related changes in local field activities, within and between cortical areas, may serve to modulate the strength of spike-based communication between cortical areas to update attention, expectancy, memory, and motor preparation during and after target recognition and speeded responding.

## Abstract 2

A new analysis considers EEG signals, not as average responses, but as individual event-related perturbations in ongoing dynamical brain processes

## Introduction

The waking brain updates and fulfills intentions through brain processes that operate within and across multiple brain areas to integrate perception, association, and action. Fulfillment of intentions is facilitated by features of these and other processes that support informed anticipation of and selective attention to events and their probable consequences. The dynamics of ongoing electroencephalographic (EEG) activity recorded from the human scalp differ markedly with state of attention and intention (Makeig and Inlow 1993; Worden et al. 2000), yet most event-related EEG research has assumed that the effects of events on EEG signals emerge out of a flat baseline, as in the typical averaged event-related potential (ERP). The electrophysiological consequences of stimulus events spread quickly in the brain. By 50–150 ms, sensory stimulus information is widely distributed (Hupe et al. 2001), perturbing ongoing patterns of local field activity in many brain areas (Klopp et al. 2000). There is little chance, therefore, that any but still earlier ERP features occur within single brain areas.

The adequacy of time-domain ERP averaging for modeling macroscopic brain dynamics also depends on the assumption that the cortical sources of EEG activity contributing to and not contributing to average ERP waveforms are somehow distinct. The scalp topographies of unaveraged EEG and averaged ERP data may, however, be quite similar (Makeig et al. 2002), strongly suggesting that areas contributing to ongoing EEG signals may also contribute to ERP averages. EEG processes not contributing to response averages may also be affected by experimental events, and several types of dynamic EEG response processes are not reflected in ERP averages (Pfurtscheller and Aranibar 1977; Makeig 1993; Makeig et al. 2002). A more comprehensive model of event-related brain dynamics is therefore needed to capture features of EEG signals that index the dynamic interplay between spatially coherent brain processes supporting anticipation of, attention towards, associations to, and behavioral responses following experimental events.

The above considerations suggest that event-related EEG dynamics may be better modeled as coordinated event-related perturbations in the statistics of multiple, intermittently active local field processes. Since the volume-conducted scalp projections of such processes generally overlap, they cannot be separately identified in the scalp-recorded data. An alternate approach we adopt here is to separate the contributions of local field processes by using distinct differences in their time courses (Makeig et al. 1996, 1997; Jung et al. 2001a).

Following stimuli belonging to an anticipated but infrequently presented category, the averaged ERP is dominated by a broad vertex-positive peak often called P300 after its earliest appearance in auditory responses (Sutton et al. 1965; for a review see Soltani and Knight, 2001). Results of ERP (Ruchkin et al. 1990) and brain lesion studies (Halgren et al. 1980; Knight et al. 1989) and of functional imaging experiments (Ford et al. 1994; Ebmeier et al. 1995; Ardekani et al. 2002) strongly suggest that P300 is actually a late positive-going response complex that sums effects of local field perturbations in several brain regions. A more inclusive model of the event-related EEG brain dynamics occurring in such data should consider, therefore, how target stimulus presentations and subject motor responses perturb the dynamics of ongoing EEG signals both within and across single subjects. Here, we present such a model.

## Results

**Figure 1:**
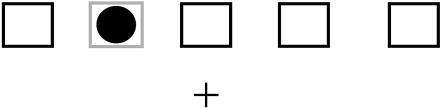
Task Display Subjects fixated on a cross above which five boxes were constantly displayed. In each 76-s task block, one of these (grey box) was colored differently. The location of this covertly attended box varied pseudorandomly across blocks. A series of disks were presented briefly in any of the five boxes in a random order. Subjects were asked to respond with a thumb button press as quickly as possible whenever a disk appeared in the attended box.

The independent component analysis (ICA) method provides a complete decomposition of single-trial (or continuous) EEG data, separating the data into distinct information sources. As the results presented below show, the amount of information about cortical dynamics provided by this method is large. Here, we detail for the first time dynamics occurring within single trials of the classes of maximally independent EEG processes whose event-related activities contribute to 31-channel visual target responses recorded during a test of spatial selective visual attention (Figure 1).

### Response Dynamics of the Scalp Recordings: ERPs and ERP Images

**Figure 2:**
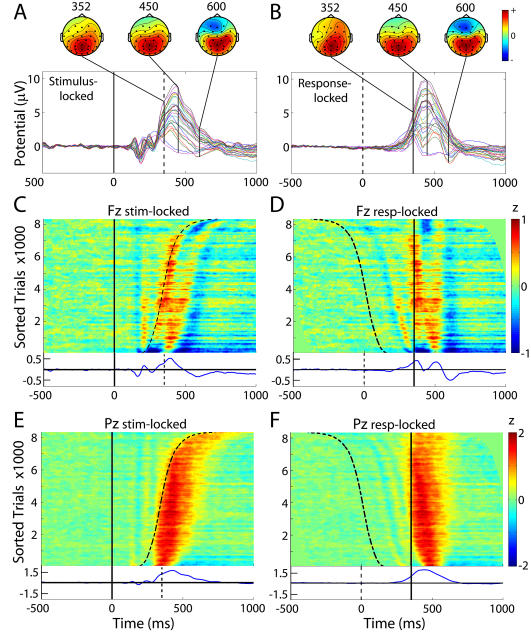
Grand Mean and RT-Sorted Single-Trial Responses at Sites Fz and Pz (Left) Stimulus-locked grand mean and (right) response-locked grand mean responses to target stimuli. (A and B) Grand mean responses at all 29 scalp channels (colored traces), plus scalp maps at indicated latencies. (C–F) Grand moving mean single-trial responses from all 15 subjects, at frontocentral site Fz (C and D) and at central parietal site Pz (E and F), plotted in ERP-image format and sorted by subject RT (curving dashed trace in left column; vertical solid line in right column, plotted at the mean subject-median response time of 352 ms). ERP-image units: z = microvolts divided by root-mean-square microvolts in the (−1000 ms to 0 ms) channel baseline EEG of the same subject after removal of eye and muscle artifact components from the data. Vertical smoothing window: 300 trials. Grand mean normalized responses are shown below each image.

To orient readers used to analyses of the raw scalp-channel data, we first present conventional ERP results at selected channels. As we previously reported, in these experiments performance level was high; more than 95% of targets were followed by a button press within the allotted response window (150–1000 ms). Mean subject-median reaction time (RT) was 352 ms. The average ERP time locked to onset of the target stimulus followed by a subject button press contained the expected late positive complex or P300 positivity following early stimulus-locked peaks conventionally termed P1, N1, P2, and N2 (Figure 2A). The scalp topography of the late positive complex varied continuously through its extent (Figure 2A, scalp maps).

In the grand average of the same epochs, each time-locked to the subject response (Figure 2B), the early response-locked peaks became smeared out, and the P300 and succeeding negative dip more concentrated. In two-dimensional “ERP-image” plots of the 8,413 single trials from all 15 subjects (Figure 2C–2F), potential fluctuations in single trials are shown as color-coded horizontal lines, here normalized by channel baseline variability then sorted (across all trials) by RT and smoothed (vertically) with a 300-trial moving average. The ERP images clearly show that the early visual response peaks at central posterior site Pz (Figure 2E) were time-locked to stimulus onset, while the late positivity at Pz immediately followed the button press (compare Figure 2E and 2F) except in the trials with the quickest RTs. Over half of these were contributed by two fast-responding subjects whose responses, unlike those of the other 13 subjects, preceded P300 onset.

At frontocentral channel Fz, however, the late positivity in the stimulus-locked grand average (Figure 2C, bottom) was largely composed of two response-locked positive peaks, separated by 200 ms, that, together with intervening and flanking negativities, could be partially modeled by a two-cycle, 5-Hz wavelet (Figure 2D). The single P300 peak at Fz in the stimulus-locked ERP (Figure 2A) “smears out” the two-cycle pattern that is captured clearly in the response-locked average (Figure 2B and 2D), while highlighting a concurrent, broader, and possibly stimulus-locked positivity in faster-RT trials (Figure 2C).

#### Event-related spectral perturbations

**Figure 3:**
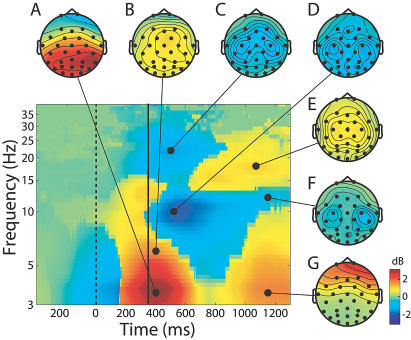
Changes in Mean Scalp Spectral Power Time-Locked to the Subject Response The solid vertical line indicates moment of the motor response (shown at the grand mean subject median RT, 352 ms). Color scale: decibel change from prestimulus baseline. Image shows signed-RMS power changes across all 29 scalp channels prior to removal of all but the largest eye artifacts. Scalp maps show the scalp topography of the spectral power change in decibels relative to baseline. Note (A and B) the broad posterior low-theta- and anterior higher-theta-band maxima at the button press, (C, D, and F) the bilateral central alpha and beta blocking, (E) the central lateral postresponse beta increase, and (G) the increase in low-frequency eye artifacts at the end of the record.

Figure 3 summarizes the grand mean time course of changes from prestimulus baseline in log spectral EEG power at all the scalp channels time-locked to button presses (solid vertical line) across the EEG frequency range. During and just prior to the button press, an approximately 3-dB increase in low-theta-band power peaked (red area) near 4 Hz in bilateral central and posterior cortex. This increase remained significant (*p* < 0.01) for 14 of the 15 subjects even after the subject-mean ERP was subtracted from each trial (data not shown).

A concurrent but weaker theta power increase near 6 Hz (Figure 3B) was maximal at frontocentral and parietal scalp sites. The theta increase at these sites was accompanied by a blocking of mu activity around 10-Hz and 22-Hz, maximal at the left and right central scalp but also widespread over posterior scalp (Figure 3C, 3D, and 3F). Following the button press, a late central bilateral increase in beta activity (maximal at 16–18 Hz) appeared (Figure 3E). Figure 3 was computed prior to performing ICA and removing eye movement artifacts. The diffuse, far frontal increase in 3–10 Hz activity that began 500 ms after the button press (Figure 3G) doubtless reflects increased subject eye activity following the target response.

### ICA Decompositions

Conventionally, characterizing the sources of ERP (Figure 2) or event-related spectral perturbation (ERSP) (Figure 3) processes is thought to be difficult because the scalp sensors are relatively far from the actual brain sources and therefore each sums the volume-conducted activities of several source areas. Moreover, the biophysical inverse problem of determining the potential source distribution from a given scalp map is in general severely underconstrained, with many mathematically correct but physiologically different solutions. Nonetheless, infomax ICA, applied to the concatenated single trials for each subject, after removing trials containing out-of-bounds or uncharacteristic artifacts, decomposed the whole set of concatenated EEG signals into 31 spatially fixed, maximally temporally independent component processes, and the scalp maps associated with many of these processes resembled the scalp projections of synchronous activity in either one or sometimes two nearly bilaterally symmetric cortical patches

#### Component contributions to the single-trial EEG signals

**Figure 4:**
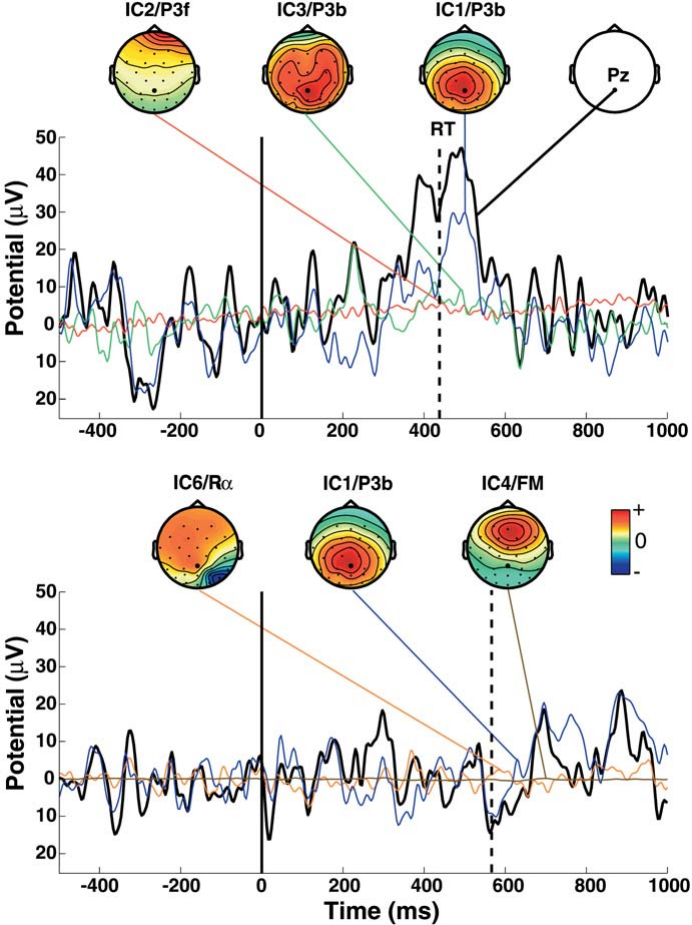
Independent Component Decompositions for Two Single Trials Black traces indicate two of 561 single target-response trials from one subject at scalp site Pz (upper right scalp map). Solid vertical lines indicate stimulus onsets; dashed vertical lines indicate button presses. A prominent late positivity occurred in the upper trial. All 561 1-sec, 31-channel EEG epochs time-locked to target stimuli were concate-nated and decomposed by infomax ICA, yielding 31 maximally independent data components. Colored traces show the projections (in microvolts) to this scalp channel of the three (nonartifact) independent components contributing the largest variance to each postresponse data window, linked to (individually scaled) maps of their scalp topographies. Component numbers (IC1–IC6), ranked by total EEG variance accounted for, and cluster affiliations (P3f, P3b, FM, P3b, Rα) are indicated above the scalp maps. Note differences in the time courses of IC1.

Figure 4 shows two single trials at site Pz (black traces) from one subject after removal of six eye and muscle artifact components. Projected activities of the three independent components most strongly contributing to each trial are shown as thin colored traces and accompanying scalp maps. Since infomax ICA provides a complete linear decomposition, the observed data (black traces) are in each case the sum of the remaining 25 (31 minus six) component projections, including the three component projections shown. In the upper trial, and typically, the single-trial P300 at Pz was accounted by ICA as summing contributions from at least two independent EEG processes. Component IC1 for this subject (ranked first by amount of total EEG variance accounted for) was later included in the parietal “P3b” component cluster (described below) on the basis of its scalp map and activity power spectrum.

While component IC1 accounted for the largest part of the P300 peak in the upper trial, in the lower trial the same IC1 process showed mixed low alpha and beta activity with a smaller postmotor response positivity. Note that the positive postresponse contribution of IC1 in this trial (thin blue trace) was sometimes larger than the observed positivity in the whole-channel data (thick black trace). At these times, some of the other 24 components contributed negative potentials to the signal at this scalp channel, partially canceling the IC1 positivity in the recorded data. Thus ICA, applied to the continuous or concatenated single-trial data, may actually recover more of the actual projected signals than are available in the single-channel data.

#### Independent component clusters

Cluster analysis, applied to the normalized scalp topographies and power spectra of all 465 components from the 15 subjects (see Materials and Methods), identified at least 15 clusters of components having similar power spectra and scalp projections. These component clusters also showed functionally distinct activity patterns. Six distinct component clusters (data not shown) accounted for eye blinks, horizontal eye movements, and left and right temporal muscle noises, respectively. These were effectively removed from the activity of the other component clusters by the ICA decomposition and are not further considered here.

#### Equivalent dipole locations

**Figure 5:**
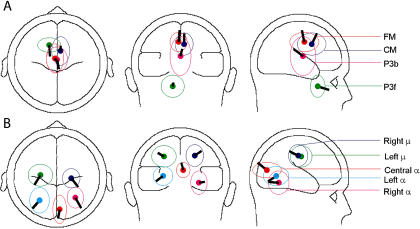
Mean Component Cluster Equivalent Dipole Locations The mean scalp map for each of the nine component clusters could be well fit by a single equivalent dipole (mean residual variance: 4.8%). The figure shows the locations and orientations of these dipoles, as determined by BESA, plotted on the spherical head model, with ellipses showing the spatial standard deviations of the locations of the equivalent dipoles for the individual components in the cluster.

Figure 5 shows the results of modeling the grand mean scalp maps for each of the nine independent component clusters as the projection of an equivalent dipole. Residual scalp map variances unaccounted for by these models were relatively small (range: 0.87% to 9.55%; mean: 4.93%), though the equivalent dipole locations for the individual clustered components were not all tightly clustered, as shown by the spatial standard deviation ellipses.

The location of the equivalent dipole for a radially oriented cortical source patch (or here, effective sum of patches) is typically deeper than the cortical patch itself (Baillet et al. 2001). The equivalent dipoles for individual components in the P3b cluster (data not shown) were scattered across parietal and central cortex (as indicated in Figure 5 by P3b's larger spatial standard deviation). Therefore, the equivalent dipole for the mean scalp map of the P3b cluster was unnaturally deep and represented the center of the active cortical source distribution only symbolically. The mean equivalent dipole location for the cluster designated “P3f” was estimated chiefly from the two periocular electrodes. Moreover, the complicated geometry of the frontal skull cannot be well fit to the spherical head model used here. Thus, the mislocalization of the P3f cluster equivalent dipole below the orbitofrontal brain surface should not be taken literally.

The spatial standard deviations of the other component cluster dipoles were smaller. Their equivalent dipoles indicate the respective dominant cortical regions of their source domains. Though the mean cluster scalp map for the central occipital alpha cluster (Cα) could be fit satisfactorily by a single equivalent dipole located in the central occiput, for several of the cluster components a better model of the component scalp map was obtained from a symmetric dipole pair in left and right pericalcarine cortex (data not shown).

### Component Cluster Dynamics

**Figure 6:**
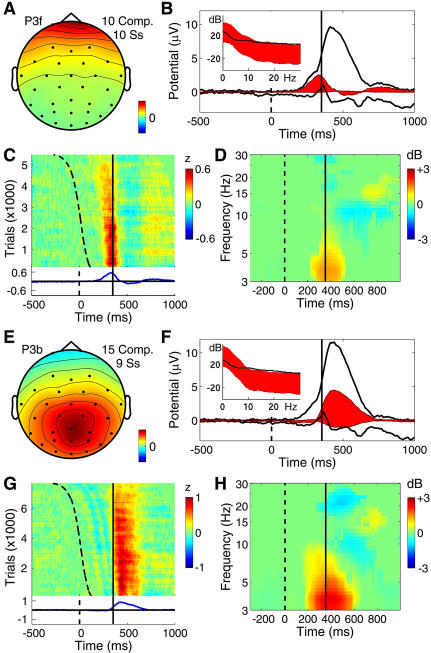
Far-Frontal and Parietal Component Clusters Contributing to the P300 (A–D) Far-frontal component cluster accounting for the preresponse (P3f) positivity. (E–H) Broad parietal component cluster accounting for part of the postresponse (P3b) positivity. The periresponse energy increase for these processes peaks at below 5 Hz. (A and E) The mean component scalp map. (B and F) The whole-data (black traces) and cluster-accounted (red fill) ERP envelopes (minimum and maximum voltage channel values at each time point), plus (inset) the power spectrum of the whole EEG (black traces) and the whole response-locked average ERP (red fill). The lower edge of the red fill shows actual ERP power, the upper edge, the phase-random EEG spectrum required to produce the observed average ERP spectrum by phase cancellation. The difference between the upper edge of the red fill and the actual EEG spectrum (black trace) reflects phase consistencies across trials in the single trial data. (C and G) ERP-image plot of the color-coded single trials time-locked to the response (solid vertical line) and sorted by RT from stimulus onset (dashed line). Trials normalized by dividing by the standard deviation of component activity in the 1-s prestimulus baseline. (D and H) The component mean ERSP showing mean event-related changes in (log) spectral power across data trials time-locked to the response (solid line). Here, median stimulus delivery time is indicated by the dashed line.

**Figure 7:**
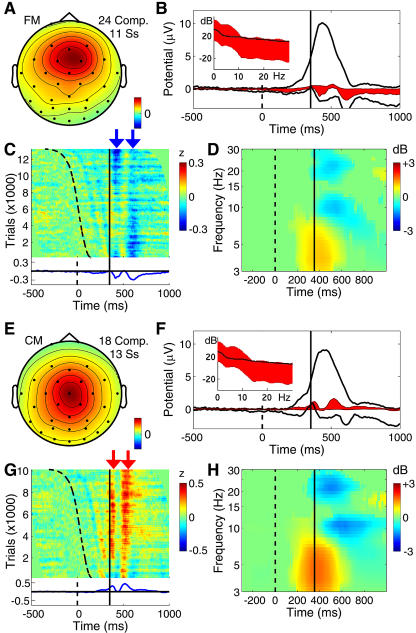
Two Mediofrontal Independent Component Clusters Showing a Postmotor Theta Response Pattern Panels as in Figure 6. (A–D) FM cluster of components often exhibiting a theta-band peak in their activity spectra. (E–H) CM component cluster projecting maximally to the vertex.

**Figure 8:**
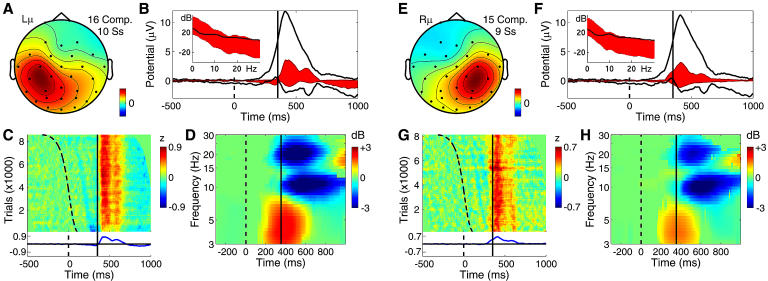
Two Mu Rhythm Component Clusters Also Showing the Postmotor Theta Response Pattern Panels as in Figure 6. (A–D) Left mu rhythm (Lμ) component cluster with mu characteristic 10-Hz and 22-Hz peaks in the activity spectrum. (D) Following the button press, this activity is blocked. (E–H) Corresponding right mu rhythm (Rμ) component cluster.

**Figure 9:**
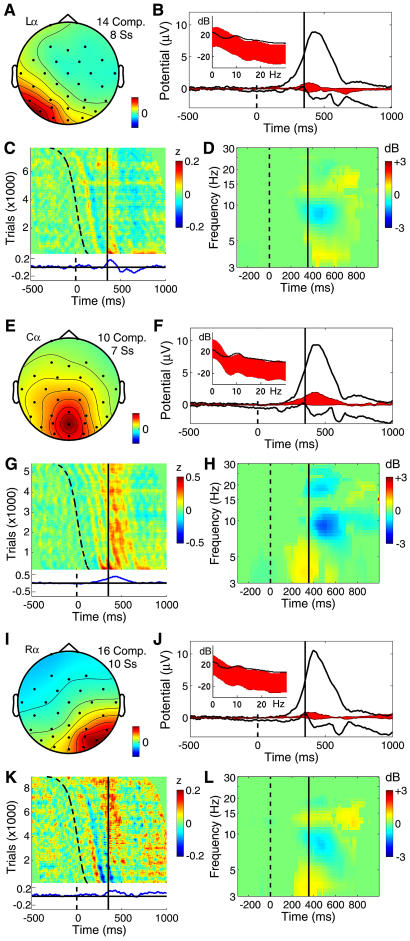
Three Posterior Alpha-Rhythm Component Clusters Panels as in Figure 6. (A–D) Left posterior alpha (Lα) component cluster. (E–H) Central posterior alpha (Cα) component cluster with characteristic trapezoidal scalp projection, consistent with a bilateral, pericalcarine equivalent dipole source model, and demonstrating prolonged phase resetting following stimulus onset (curving dashed trace). (I–L) Right posterior alpha (Rα) component cluster. Note (C) the relative absence of the alpha-ringing pattern in the Lα cluster activity and the (D, H, and L) relatively weak postresponse alpha blocking in these clusters.

Mean dynamics properties of nine nonartifact component clusters are summarized in Figures 6–9, each of which shows the mean scalp map and response-locked ERP image, activity and ERP spectra, and ERSP for one or more component clusters. Because of the complexity of the results, we report and interpret the nine component clusters in four groups based on shared dynamic features.

#### Two clusters contributing to the late positive response (P3f and P3b)

Two component clusters made distinct contributions to the late positive complex of the target ERP. After subtracting the larger back-projected scalp-data contributions of components accounting for blinks and saccadic eye movements, the response-locked ERP at both periocular channels contained a broad, approximately 2-μV positive-going scalp potential peaking on average 39 ms before the recorded button press. Figure 6A–6D shows the mean scalp map and dynamic properties of a cluster of ten independent components from ten subjects that together largely accounted for an ERP feature whose time course was highly similar to the peak we labeled P3f (for P3-frontal) in an earlier report on decomposing the matrix of 25 condition ERPs from these experiments (Makeig et al. 1999a). Note, in the ERP image (Figure 6C), the absence of sharp excursions not regularly time-locked to experimental events, which would mark blinks or lateral eye movements. Such activity at far frontal and periocular channels was effectively separated out by ICA into artifact components (data not shown). Instead, as shown in Figure 6B, the P3f component cluster accounted for nearly all the positivity occurring before the button press (designated P3f by Makeig et al. 1999a), particularly in shorter latency-response trials (Figure 6C).

The P3f cluster-mean response-locked positivity began near 150 ms, consistent with direct neurophysiological evidence that by 150 ms after stimulus onset, visual information is spread throughout the brain by a complex web of afferent and efferent connections (Klopp et al. 2000; Hupe et al. 2001). Subtracting the button travel time (approximately 25 ms, roughly estimated from electromyographic recording during one experimental session) and the neuromuscular conduction time (approximately 15 ms) suggested that the P3f peak at 39 ms before the button press occurred very near the moment of the subcortical motor command. It is thus tempting to speculate that the P3f process should originate in frontal structures involved in motivated decision making and response selection, such as orbitofrontal cortex (Ikeda et al. 1996), though the sparse spatial sampling of the present data does not allow more specific conclusions.

The far-frontal (P3f) component cluster response appears similar to the 280-ms “P2a” peak noted in responses to (foveal) visual “oddball” stimuli by Potts et al. (1998). Potts and Tucker (2001) reported that P2a was maximal near the eyes but can be recorded over most of the face and may also be found in attention conditions involving no subject button press. The scalp map of the ERP-derived P3f component derived by ICA applied to the 25 condition ERPs (Makeig et al. 1999a) also included bilateral parietal features not seen here in the P3f component cluster. Evidently, the temporal and far-frontal projections joined in the ERP-derived P3f component map did not cohere in the much more extensive single-trial data and so were separated by ICA applied to the concatenated single-trial data. This detail points to the advantage of decomposing a sufficient number of unaveraged data trials over decomposing even a relatively large set of averaged responses.

Figure 6E–6H shows the mean scalp map and activity patterns of a bilaterally distributed cluster of 15 components (from nine subjects) that projected most strongly to posterior and central scalp sites and made a substantial contribution to the slow postmotor P300 or P3b positivity. Examination of the raw ERP waveforms of the six subjects not contributing to this cluster suggested the absence of a typical central parietal positivity in their target responses. The mean cluster scalp map (Figure 6E) resembled that of the response-locked parietal ERP peak itself (see Figure 2B). The ERP image of the normalized single-trial component activity (Figure 6G) includes an early series of small, positive and negative, wave fronts following the (sorted) time of stimulus delivery (dashed curve) by fixed delays. These are followed by a large response-locked positivity (red area) accounting for 62% of postre-sponse ERP variance at 300 ms for site Pz. The P3b cluster positivity is clearly smaller in late-response trials (Figure 6G, top), consistent with the Pz data (Figure 2F). The mean cluster ERSP (Figure 6H) reveals a significant (3-dB) post-response low-theta power increase.

The significance of the stimulus-locked P300 (or P3b) peak over central parietal cortex has long been debated. Our results clearly show (Figure 2E) that in these experiments, this peak was time-locked to and predominantly followed the motor response. P3b onset occurred at about the moment of the motor command, coincident with the P3f peak. It could not, therefore, index activity involved in making the motor decision or action, as this is seemingly associated with the P3f process. The equivalent dipole distribution of the P3b cluster was broad, the strongest commonality being dipole orientation toward the central parietal scalp. Between-subject variability in locations of P3b generators have also been reported by researchers using other source localization methods (Moores et al. 2003). It is possible that more advanced three-dimensional component clustering methods, applied to decompositions from more subjects and more data channels, might allow further distinctions among processes in this cluster.

#### Central midline clusters

Figures 7 and 8 show mean properties of four classes of components producing the two-cycle postresponse evoked-response pattern seen clearly in the response-locked data at site Fz (see Figure 2D). Principal among these were two component clusters (Figure 7) projecting maximally to the frontal midline (FM) and central midline (CM) scalp, respectively.

In the RT-sorted FM cluster ERP image (Figure 7C), the two negative wave fronts follow the curving trace marking stimulus onsets, the second of these merging with the earlier RT-locked negativity. Though the vertex-maximum CM component cluster (Figure 7E–7H) also exhibited the postmotor theta feature (Figure 7F and 7G, red arrows) with the mid frontal and mu rhythm clusters (see below), it contributed little to the broader (P3b) positivity produced mainly by the central parietal (see Figure 6E) and central occipital (Figure 9) component clusters.

Though the equivalent dipole locations of components in the two midline clusters were somewhat overlapping (see Figure 5), their mean equivalent dipole locations were generally consistent with sources in or near the dorsal anterior cingulate and cingulate motor areas, respectively (Ullsberger and von Cramon 2003). These areas are implicated by fMRI and neurophysiological experiments as participating in motor response selection and anticipation of the consequences of events, including those involving self-perceived errors (Shima and Tanji 1998; Luu and Tucker 2001; Manthey et al. 2003; Ullsperger and von Cramon 2003). The phase/latency of the postmotor theta burst appeared to be consistent across quicker and slower responses.

The theta burst appears to resemble other reported FM EEG activity patterns: theta bursts or trains (fmθ) appearing during mental concentration (Mizuki et al. 1980; Gevins et al. 1997; Uchida et al. 2003) and brief bursts of theta activity linked to and following the error-related negativity (Luu and Tucker 2001; Luu et al. 2004), an ERP peak whose latency matches the first negativity in the FM cluster postresponse ERP (at approximately 60 ms). Inverse source modeling has placed the generating cortical domain of the ERN and fmθ in or near the dorsal anterior cingulate. In this ICA decomposition, however, the two-cycle postmotor theta burst pattern appeared not only in the FM cluster, but also in the CM, mu, and parietal clusters (see below).

The scalp map of the CM cluster (Figure 7E) resembles scalp maps of the “P3a” or “P3novel” ERP peaks seen, e.g., when unique and unexpected stimuli are included in a randomly alternating sequence of target and nontarget stimuli (Courchesne et al. 1975; Polich and Comerchero 2003). Here, however, the CM cluster made only a small contribution to the stimulus-locked target ERP.

#### Mu rhythm clusters

The left and right mu rhythm component clusters (Lμ and Rμ in Figure 8) exhibited the defining feature of mu rhythms—distinct spectral peaks near 10 Hz and 22 Hz that are strongly blocked following movements, with equivalent dipoles located roughly over hand motor cortex (and/or adjacent postcentral somatosensory areas), and oriented roughly orthogonal to the directions of the central sulci. Both the ERP and ERSP peaks were larger in the left mu cluster (contralateral to the response hand) than in the right. In common with the midline clusters, the mu component clusters contained the two-cycle postmotor theta pattern (Figure 8D) concurrent with a mean theta power increase (Figure 8H). They also made slower, positive-going contributions to the parietal ERP, particularly to the late “slow wave” phase of the stimulus-locked P300 complex that, unlike the main (P3b) peak, exhibits a polarity reversal over the central scalp (Simson et al. 1977).

In a previous ICA analysis of ERPs from these experiments, the late slow wave phase of the stimulus-locked P300 complex was confined to a single component (Makeig et al. 1999a), but here it was separated into distinct left and right mu rhythm processes in at least ten of the subjects. More detailed source analysis of magnetoencephalographic mu rhythms has assigned their source mainly to somatosensory cortex (Forss and Silen 2001). Thus, the postresponse slow positivity (Figure 8C) of the Lμ cluster, larger following slower responses, might index tactile feedback from the hand and button surface (Makeig et al. 1999a).

#### Posterior alpha clusters

Figure 9 shows the dynamics of three clusters of components projecting to the posterior scalp. Each had a distinct near 10-Hz alpha frequency peak in its activity spectrum, most pronounced in components of the central cluster (Figure 9F, inset). The stimulus-locked ERP contributions of the two lateral posterior alpha clusters, shown as sloping wave fronts in Figure 9C and 9K, included an early stimulus-locked peak accounting for most of the P1 ERP peak (near 145 ms) and for part of the succeeding N1 peak, which summed contributions from several clusters. In the central alpha component cluster, the initial stimulus-locked response feature was followed by a train of approximately 10-Hz stimulus-locked waves. These can be said to be produced by partial phase resetting of the intermittent alpha activity of these components following stimulus onsets, since they were accompanied by *no* mean increase in alpha power. The central alpha cluster also made an appreciably broad, triangular contribution (Figure 9F and 9G) to the P300 positivity, while the contributions of the lateral clusters to the response-locked ERP, beginning just before the button press, were small and narrow. The mean response-related ERSPs for these three clusters were weak (Figure 9D, 9H, and 9L), and their postresponse alpha and beta blocking were brief and weak, compared to the two mu clusters (see Figure 8). The lateral clusters, but not the central cluster, exhibited a low beta increase above baseline (near 14 Hz) beginning near the button press.

While the existence of multiple alpha rhythms has long been noted, ICA here neatly separated their activities and identified their complete, overlapping scalp maps based on the relative independence of their activity patterns in the unaveraged data. The central posterior alpha processes had a stronger alpha-band peak than lateral posterior alpha components, and showed longer-lasting phase resetting following visual stimulus onsets (Figure 9G). The longer phase memory implied by the prolonged phase resetting is compatible with longer bursts of alpha activity in these components. Possibly, the distinct dynamics of the central and lateral posterior clusters may serve different though still unknown purposes.

The “trapezoidal” signature of several of the central posterior component scalp maps (Figure 9E) is compatible with a model comprising two equivalent dipoles located symmetrically in left and right pericalcarine cortex. To be fused into a single infomax ICA component, activity in both hemispheres must have been largely synchronous with negligible phase delay. Alpha-band activity in two cortical areas can indeed be synchronous if the two areas are densely connected, here most likely via corpus callosum. Synchronization of bilateral generator regions via dense callosal coupling might also support the observed sharp (around 10-dB) alpha peak in the activity spectra of these components.

In these data, the lateral posterior alpha components were always unilateral and never bilateral. Possibly this may reflect the lower density of direct connections between these areas. Early ERP features in these experiments appeared predominantly contralateral to the stimulus locations. No doubt this was because these stimuli were presented above and usually lateral to fixation. In other data, we have noted that visual ERPs time-locked to foveally presented stimuli usually contain a bilateral posterior P1/N1/P2 complex (Makeig et al. 2004).

### Component ERP Contributions

**Figure 10:**
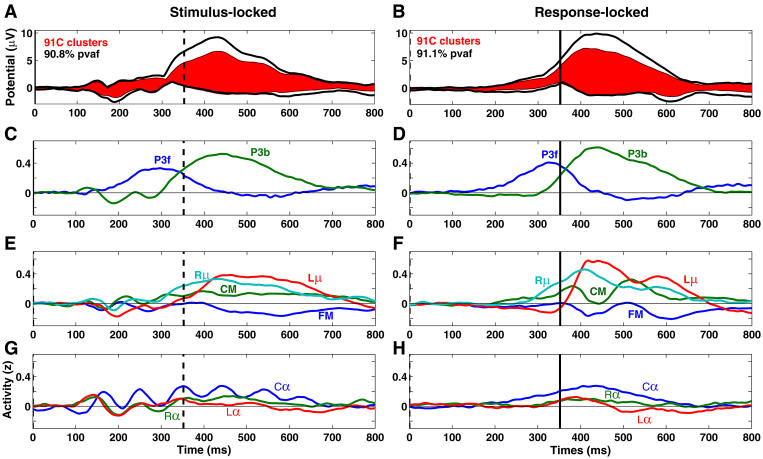
Component Time Courses and Summed Scalp Projections Summed projections (A and B) to the grand mean ERP average of all trials time-locked to stimulus onsets (left) and to subject responses (right), plus (C–H) grand mean normalized activity time courses of each of the nine independent component clusters, scaled and separated into the same cluster groupings as in Figures 6–9. For comparison with the stimulus-locked responses (left), response-locked data epochs (right) are shown aligned to the mean subject-median response time (352 ms, dashed line in left panels).

Together, the nine component clusters accounted for 91.1% of the variance of the response-locked grand mean ERP at all channels in the 1000 ms following stimulus onset, as well as for 90.8% of the variance of the stimulus-locked grand mean ERP. Figure 10A and 10B shows the envelopes (most positive and negative channel values, across all channels, at each time point) of the stimulus-locked and response-locked grand mean ERPs (black) and the envelope (red fill) of the summed back-projections to the scalp of the components comprising the nine clusters. The normalized grand average activity time courses for the nine clusters are shown in Figure 10C–10H, for comparison with the time courses of the grand mean ERP (Figure 10A and 10B).

Note that stimulus-locked component cluster ERP activity first appeared in the lateral posterior alpha clusters (at 100 ms). Onset of the stimulus-locked ERP of the P3b cluster at about the same time was soon followed by the far-frontal P3f cluster onset (near 120 ms, Figure 10C). The stimulus-locked ERP deflection began at the same moment in the four postmotor theta clusters (Figure 10E). Six of the nine clusters had a negative peak in their stimulus-locked ERP average near 200 ms, confirming the spatial complexity of the N1 peak, as indicated by invasive measures (Klopp et al. 2000) and comparable to previous analysis of nontarget epochs from this data set (Makeig et al. 2002).

In the response-locked cluster ERPs, note that the P3f cluster activity appeared to begin early, while response-related activity in the P3b cluster ERP diverged from baseline 10–20 ms before the P3f peak, concurrent with a posterior-negative peak in the left mu cluster ERP. The posterior-positive peaks in the response-locked ERPs of both mu clusters, the early shoulder of the P3b cluster ERP peak, the central cluster ERP slow wave, and the negative-going peak of the FM cluster ERP all occurred together, about 100 ms after the P3f peak.

**Figure 11:**
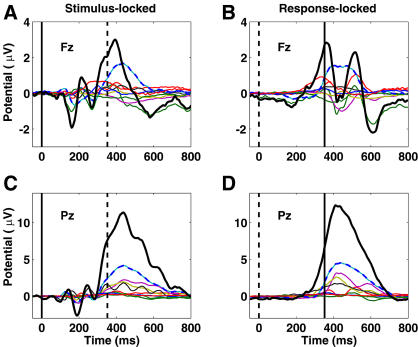
Cluster Projections to the Scalp ERP Component cluster contributions (in microvolts, thin traces) to the grand mean stimulus-locked (left) and motor-response-locked (right) target ERPs at scalp sites Fz (top) and Pz (bottom), plus their summed contributions (thick traces). Although the P3b cluster makes the largest contribution to the evoked responses at both scalp sites, its contribution does not outweigh the summed contributions of the other clusters.

Figure 11 shows the individual and summed independent component cluster contributions to the grand mean ERP at sites Fz and Pz. At Pz, no component cluster contributed more than a third of peak parietal P300 amplitude in either the stimulus-locked or response-locked ERPs (Figure 11C and 11D). The largest cluster contribution to the peak at Fz was also from the P3b cluster, which contributed about half its peak amplitude (Figure 11A and 11B). The P3f cluster contributed at best a third. These results cast doubt on claims that the target-response P300 peak at Fz predominantly indexes frontal activity.

### Post-Response Theta Synchronization

**Figure 12:**
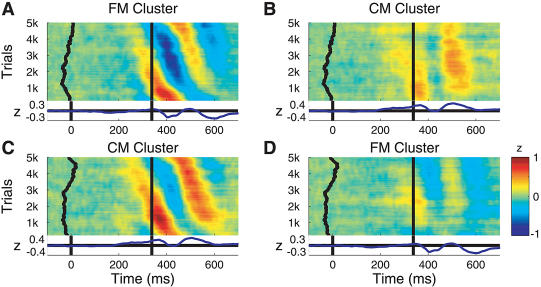
Phase Coupling of Theta Components: Time-Domain View (A) ERP-image view of baseline-normalized, response-aligned single-trial activity time series of components in the FM cluster, sorted (top-to-bottom) by phase at 4.87 Hz in a window centered 89 ms after the button press. Vertical smoothing: 400 trials. Units: microvolts normalized by dividing by the standard deviation of component single-trial baseline activity. The curving vertical trace (left) shows a moving mean of stimulus onset times; the central vertical line, the time of the button press. Data band pass in all panels: 0.1–40 Hz. (B) Exporting the same trial sorting order from (A) to CM cluster components (from the nine subjects contributing components to both clusters) demonstrates the significant partial theta phase coherence (r is approximately 0.3) between the two clusters in the postresponse time/frequency window. Note the induced (top-down, left-to-right) slope of the latency of the two (orange) positive-going CM cluster theta wave fronts. (C) Phase-sorted ERP image, as in (A), of the normalized CM cluster trials. (D) FM cluster component trials sorted in the same trial order as (C). Again, the partial theta-band phase coherence of the two clusters in the postresponse period is reflected in the diagonal (blue) negative-going wave fronts of the FM cluster data.

Phase coherence analysis of consistent phase relationships between the FM and CM clusters, and between the FM and left mu clusters, time-locked to and following the motor response showed that significant theta phase coherence appeared in the data, even after the respective component ERPs were subtracted from each trial, indicating a transient postresponse phase linkage between these otherwise maximally independent processes. Figure 12 illustrates this phenomenon in the time domain using phase-sorted ERP-image plots. Sorting trials by the phase (with respect to the button press) of the postmotor theta activity of the FM cluster (Figure 12A) and then imaging the single-trial activities of the CM cluster in the *same* trial order (Figure 12B) induced partial theta phase ordering on the CM data (i.e., the slightly diagonal wave fronts in Figure 12B). The converse procedure (Figure 12C) gave a similar result (Figure 12D).

Video 1 is an animation representing the joint response-related theta-band dynamics occurring in and between all nine component clusters (Delorme et al. 2002). The figure shows an analysis window centered 89 ms after the button press. Note that the transient theta phase coherence between the mu, parietal, and midline components was selective: phase coherence between FM and CM, FM and Lμ, and Rμ and P3b clusters (indicated by linking cylinders) were significant, whereas no significant phase linkage occurred in this time period between the FM and P3b clusters, nor between the CM and Lμ clusters. This selectivity diminishes the possibility that the observed transient phase linkages were produced by appearance of postresponse EEG activity not separated out by ICA into a separate component and therefore misattributed by the ICA model to nearby independent components. The results presented here, however, do not allow us to completely discount this possibility.

## Discussion

ICA used the temporal information contained in the single-trial EEG time courses to identify and separate maximally independent processes. These were associated with overlapping scalp maps and time courses whose distinctive features were no longer blurred by volume conduction as in the scalp electrode data. The nine independent component clusters here identified by their similar scalp projections and activity spectra resemble classes of EEG phenomena long described by neurologists from observation of paper data displays—central and lateral alpha, left and right mu, and FM theta rhythms. By cleanly separating the EEG contributions of these processes, ICA allowed exploration of their individual and joint event-related dynamics. Our finding of selective theta synchronization between FM, motor, and parietal processes (Video 1) was only possible using ICA. The clear separation of “alpha ringing” in the stimulus-locked response from the other ERP features (see Figure 10G) also illustrates the power of ICA to separate temporally and functionally distinct activities that are generated in different brain areas but project to the same scalp channels.

The nine clusters largely reproduced the component clusters we obtained previously from ICA decomposition of brief (100-ms) poststimulus time windows following nontarget and target stimuli in these experiments (Makeig et al. 2002). The major difference in the two sets of clusters was the inclusion, here, of a parietal P3b component cluster. In both analyses, the clustering omitted many small components with “noisy” scalp maps and time courses, and also omitted “outlier” components specific to single subjects. After removing clear ocular and muscle artifact components from the raw data, however, the nine identified EEG component clusters together accounted for over 90% of the grand mean ERP variance (over all channels) as well as almost 60% of variance in the whole EEG. By contrast, the ERP data themselves accounted for only 6% of poststimulus EEG variance, supporting our claim that this analysis presents a more complete model of the event-related EEG dynamics occurring in these data than the averaged ERP.

### Cluster Localization

The group mean equivalent dipole locations shown in Figure 5 only symbolize the actual distribution of the component source domains. The relation between processes derived by ICA from scalp data and processes seen in invasively recorded cortical data are not yet clear. For example, the equivalent dipole localization of the P3b cluster-mean scalp map does not correspond directly to all the cortical areas noted to generate P3b-like local field potentials in implanted presurgical epileptic patients by Halgren et al. (1995a, 1995b)—parts of the superior parietal lobule, inferior frontal, and temporal cortices, as well as the limbic medial temporal lobe. However, in general it is difficult to infer the cortical distribution of a cortical source domain from equivalent dipole location. Clearly, more inclusive methods of ICA component source localization fitted to actual subject cortical geometry (Dale and Sereno 1993) may be useful for further research. It is not clear, however, whether “hot spots” recorded by sparsely implanted intracranial electrodes necessarily map activity that dominates the scalp EEG dynamics; hot spots might arise from focal or more spatially diffuse activities in other areas.

### ERSPs

Production of scalp EEG signals requires partial synchronies in local field activity extending across the relatively large (2-cm or more) domains of neuropile (Nunez 1981). These must be dynamically maintained, and might well be perturbed by biological systems and mechanisms that implement and reinforce “top-down” cognitive decisions such as, as here, to selectively attend and respond to relatively infrequent visual stimuli while ignoring frequent nontargets (Giesbrecht et al. 2003). Posterior alpha, in particular, increases promptly when visual attention shifts between hemifields or between visual and auditory stimulus streams (Worden et al. 2000). Most of the spectral perturbations appearing in this analysis have been previously reported in some form, for instance, the alpha blocking following visual stimuli cueing visual attention and mu blocking accompanying cued finger movements (Hari et al. 1997; Pfurtscheller et al. 2000). A late beta increase following target responses has also been reported (e.g., Makeig 1993).

### ERP Influence

Traditionally, averaging event-locked EEG data from single scalp channels to form an ERP is assumed to reject, by random phase cancellation, “background” EEG rhythms whose statistics are tacitly assumed to be unaffected by experimental events. To test the effect of the average ERP on the observed spectral perturbations, we computed the cluster ERSPs again after removing the component mean ERP from each trial (data not shown). All the effects shown in Figures 6–9 remained significant.

### Common ERSP Features

Note that many of the cluster ERSPs (Figures 6–9) share common features. How is this possible? First, “independent” components returned by infomax decomposition of EEG data are never perfectly independent, but are instead those found by infomax to be maximally independent. This is not a mere play on words, but an advantageous feature of infomax decomposition that allows it to separate activity from different cortical areas even when the independence of synchronous activities within those areas is not unbroken or absolute. Second, the infomax independence metric is weighted toward separation by phase differences rather than by power spectral differences. The observed spectral perturbations may reflect, in part, common modulatory influences of central neurotransmitter-labeled brainstem systems involved in orienting and arousal, which project widely to cortex and are known to change the spectral properties of cortical field activity following novel or meaningful events (Aston-Jones et al. 2001; Fries et al. 2001; Hasselmo et al. 2002).

### Functional Significance of the Postmotor Theta Bursts

At and after the button press, a mean frontocentral theta power increase appeared in all 15 subjects' data. It was partially phase coherent in four of the component clusters and was not eliminated by removing the subject-mean ERP from each trial. Local bursts of theta-band activity are widely distributed on the human cortex (Kahana et al. 1999) and associated with cognitive function (Caplan et al. 2003). In hippocampus, an association between theta phase and high-frequency “sharp wave” activity has been observed in nonhuman animals (Csicsvari et al. 2003). In turn, high-frequency activity can index the organization of spike timing of similarly tuned neurons into brief near-synchronous volleys more likely to trigger further spikes in common target neurons (Fries et al. 2001; Salinas & Sejnowski 2002).

Following nontarget stimuli in this experiment, FM components exhibited weak “theta ringing” (partial poststimulus theta phase locking) not accompanied by increased theta power (Makeig et al. 2002). Here, following targets, a two-cycle period of increased theta activity appeared, time-locked to the motor response and weakly phase coherent between FM, parietal, and motor areas. Coherent theta activity might enhance the speed, salience, and reliability of spike-based communication between these and other brain areas connected with them, including hippocampus and related limbic structures such as the amygdala (Seidenbacher et al. 2003). The result might be facilitation of information transfer to and from memory structures about the event and its anticipated consequences, and selective retuning of attentional states in relevant cortical areas based on anticipatory evaluation of the consequences of the cued motor response, including readjusted sensory and motor expectancies.

The postmotor theta bursts seen here following correct speeded responses very likely are tightly linked to the ERP feature with strong theta-band energy that follows highly speeded manual (or foot) responses in the Erickson flanker task (Holroyd et al. 1995). Luu and Tucker (2001) have suggested that the so-called error-related negativity in the response-locked ERP following responses the subject knows immediately to be in error partly represents partial phase locking of transiently increased theta-band activity in FM and other sources (Luu et al. 2004). A similar negative-going ERP feature has been reported following negative feedback whose valence is not known in advance (Gehring and Willoughby 2002; Ruchsow et al. 2002). Luu and Tucker (2001) also reported the appearance of enhanced theta activity in the ERP above somatomotor cortex following known-error responses. Our animation of the event-related theta-band dynamics in our data (see Video 1) demonstrates that correct speeded button presses produce partially synchronized theta-band increases in frontal (but not central) medial and contralateral somatomotor process clusters. Mean coherence phase lag between the midline clusters suggests that the postmotor theta activity in the FM cluster components leads that of the CM components by about 8 ms, a physiologically plausible value whose statistical reliability should be tested on a larger data set.

### Postmotor Theta and P300

Elbert and Rockstroh (1987) have proposed that cortical surface positivities in general index periods of relative neural depolarization and concomitant insensitivity (“disfacilitation”) of the involved cortex to distal input, possibly explaining the concurrent attentional blink (McArthur et al. 1999; Kranczioch et al. 2003) and amplitude decrease in the auditory steady-state response (Rockstroh et al. 1996). von Stein et al. (2000), on the other hand, have reported that following delivery of visual targets to cats, coherence in the theta band occurred between the output layer of a higher and the input layer of a lower visual cortical area. They did not observe this coherence following nontarget stimuli. Thus, the postmotor P300 and the theta burst response may have complementary functions: to decrease bottom-up environmental sensitivity and to concurrently apply the results of top-down processing to cortical perceptual areas. Although probably not available for observation in scalp data, the postmotor theta-band synchronization might extend to limbic areas (hippocampus, amygdala, and others) and play a role in memory updating (e.g., of learned/remembered “context”) following goal-directed actions (cf. Seidenbecher et al. 2003). Thus, we suggest that the postmotor theta burst may reflect directly “context-updating” processes previously proposed to be indexed by the broad P300 positivity (Dien et al. 2003).

### Evoked Responses

In this speeded response paradigm at least, the ERP P300 positivity was nearly strictly time-locked to and predominantly followed the motor response. The P300 positivity was, here, indeed a late positive complex of potentials generated in several brain areas, confirming results of invasive recording (Smith et al. 1990) and clinical group-difference studies (Potts et al. 1998). ICA decomposed the unaveraged EEG signals composing the target-response ERP into several classes of brain EEG processes originating predominantly in frontal, central, parietal, and occipital cortex. This result adds to longstanding doubts about the specificity of ERP peak measures. In particular, it shows that parietal sources may account for less than half of the peak amplitude of the stimulus-locked positive peak at Pz (see Figure 11C), the most commonly used index of P300 magnitude. Altogether, we found four component clusters contributing to the P300 maximum at Pz—in descending order, central parietal, left and right mu, and central occipital alpha EEG processes. As well, the (3-fold) largest EEG contributor to the stimulus-locked P300 positivity at Fz is volume-conducted from the same parietal (P3b cluster) sources, casting strong doubt on the specificity of peak amplitude at Fz for indexing frontal function.

### Phase Resetting

As in our previous single-trial analysis of some of these data (Makeig et al. 2002), partial phase resetting of ongoing intermittent alpha and theta EEG processes contributes to some features of the average ERP. However, partial phase resetting is not a sufficient model for all the ERP features. For example, the central posterior alpha cluster actually showed a postmotor response decrease in alpha power during its prolonged alpha-ringing ERP contribution (see Figure 9G and 9H), whereas the monopolar parietal P300 (or P3b) ERP feature was associated with an event-related increase in both whole EEG variance in the central parietal channel (Pz; data not shown) and in component activity variance of the P3b, Cα, and Lμ clusters that contributed the most to it.

The central posterior alpha-ringing feature of the stimulus-locked ERP can be parsimoniously described as arising through alpha phase resetting. The P300/P3b feature, on the other hand, might better conform to the usual conception of an ERP as measuring evoked response activity added to ongoing EEG activity. Note in Figure 6H, however, that the low-frequency energy of the P3b cluster component activities increased by less than 3 dB at 3 Hz, demonstrating that the “baseline” activity of these processes included slow-wave processes with similar spectral characteristics. The postmotor theta burst phenomenon comprised both a frequency-specific power increase and significant (though partial) phase locking through its two-cycle ERP duration.

The variety of these ERP features suggests that assuming a strict dichotomy between evoked and phase-reset activities is unproductive. Rather, each ERP feature may be usefully and better characterized as summing event-related perturbations of various sorts in the ongoing activities of one or more localized cortical EEG processes. To produce a reproducible peak or peaks in the average ERP, these perturbations should involve some degree of (partial) phase locking of the contributing process activities to the time-locking events in one or more frequency regions. At very low (near-DC) frequencies, event-related “phase locking” implies event-related “sign locking.” For example, the P300 would never appear in ERP averages at all if it were negative-going in half the trials. Similarly, there need be no strong dichotomy between evoked phenomena that involve partial phase locking and so contribute to average ERPs, and induced phenomena that may involve changes in spectral amplitudes but do not show phase locking and thus do not contribute to average ERPs. Rather, it is important to realize that most event-related EEG dynamics have *both* induced and evoked aspects.

### Event-Related Brain Dynamics

The results presented here confirm that extensive, complex, and flexible information concerning links between cognitive processes and macroscopic brain dynamics are available in noninvasive high-density EEG data. Availability of more comprehensive analysis techniques, such as that introduced here, should make EEG (and related magnetoencephalographic) data analysis of increasing interest both to cognitive neuroscientists and to neurophysiologists, as event-related EEG dynamic models complement observations of slow-changing hemodynamics while greatly expanding the restricted spatial information available from single- and multineuron spike recordings.

## Materials and Methods

### 

#### Task design

ERPs were recorded from subjects who attended to randomized sequences of filled disks appearing briefly inside one of five empty squares that were constantly displayed 0.8 cm above a central fixation cross (see Figure 1) following Townsend and Courchesne (1994). The 1.6-cm square outlines were displayed on a black background at horizontal visual angles of 0 ° ± 2.7 ° and 0 ° ± 5.5 ° from fixation. During each 76-s block of trials, one of the five outlines was colored green and the other four blue. The green square marked the location to be attended. This location was varied in random, counterbalanced order across blocks. In each block, 100 stimuli (filled white disks) were displayed for 117 ms within one of the five empty squares in a pseudorandom sequence with interstimulus intervals of 250 to 1000 ms (in four equiprobable 250-ms steps).

#### Subjects and task

Fifteen right-handed volunteers (ages 19 to 53 y, mean = 30; 12 male, three female) with normal or corrected-to-normal vision participated in the experiment. Subjects were instructed to maintain fixation on the central cross while responding only to stimuli presented in the green-colored (attended) square. Subjects were required to press a thumb button held in their right hand as quickly as possible following stimuli presented in the attended location (see Figure 1). Thirty blocks of trials were collected from each subject, yielding 120 target and 480 non-target trials at each location. Subjects were given approximately 2-min breaks between blocks.

#### EEG recordings

EEG data were collected from 29 scalp electrodes mounted in a standard electrode cap (Electro-Cap International, Eaton, Ohio, United States) at locations based on a modified International 10–20 System, and from two periocular electrodes placed below the right eye and at the left outer canthus. All channels were referenced to the right mastoid with input impedance less than 5kΩ. Data were sampled at 512 Hz with an analog pass band of 0.01–50 Hz. To further minimize line noise artifacts, responses were digitally low-pass filtered below 40 Hz prior to analysis. Trials containing electrooculographic potentials larger than 70 μV or amplifier blocking were rejected, and brain responses to stimuli presented at each location in each attention condition were stored separately. Responses to target stimuli were analyzed only when (as in nearly all cases) subjects responded 150–1000 ms after target onset. The few targets followed by no such button press were not considered in the present analysis.

#### Previous analysis

Analysis of average ERP and some single-trial data from these experiments have been reported previously. Makeig et al. (1999a) first reported ICA decompositions of late (P300) target responses in a 5 × 5 matrix (five stimulus locations by five attended locations) of grand mean visual stimulus ERPs from these experiments. They reported three maximally independent ERP components of interest which they labeled P3f, P3b, and Pmp. Makeig et al. (1999b) applied the same multiple-ERP analysis to the first 250-ms period following stimulus onsets and demonstrated that distinct contributions to the N1 ERP peak were generated in the right hemisphere 9 ms earlier, on average, than in the left. ICA separates component processes mixed in scalp data based on their relative temporal independence, which should be maximally expressed in the *unaveraged* data. Systematic application of ICA to unaveraged data from these experiments was first demonstrated for non-target stimulus trials (Makeig et al. 2002). In that analysis, a 100-ms poststimulus period (150–250 ms after stimulus onset) was extracted from each of the over 3,000 trials for each subject, and these data were concatenated and decomposed by ICA. Some information about the target stimulus trials was also presented in Jung et al. (2001b). Here we report the results of comparing ICA decompositions of roughly 600 1-s target-response trials from each of 15 subjects.

#### Independent component analysis

Infomax ICA (Bell & Sejnowski 1995; Makeig et al. 1996) is one of a family of algorithms that exploit temporal independence to perform blind separation of underlying data sources. Lee et al. (1999) have shown that these algorithms have a common information-theoretic basis, differing chiefly in the form of distribution assumed for the sources, which may not be critical (Amari 1998). Infomax ICA finds, by natural gradient ascent, a square “unmixing” matrix that maximizes the joint entropy (Cover and Thomas 1991) of a nonlinearly transformed ensemble of zero-mean input data vectors. Maximizing joint entropy implies, under reasonable assumptions (Bell and Sejnowski 1995), minimizing mutual information among the component activities. This means that information about the simultaneous activity values of any number of the components gives minimum information about the concurrent activity values of any other components.

Independent component activities are minimally correlated, both in standard second-order and in higher-order senses. That is, they each appear to ICA to be “free-running” and in this sense act as separate sources of information in the data. The power of infomax source separation in a growing range of signal processing applications derives from its basic aim to identify *information sources* in data, in contrast to previous root-mean-square estimation methods that aim simply to model data *variance* (Jung et al. 2001a). Natural-gradient logistic infomax ICA in the automated form we use here (the runica algorithm, Makeig et al. 1997) can accurately decompose mixtures of component processes having symmetric or skewed distributions without requiring nonlinearities specifically tailored to them, and can be usefully applied to EEG data from 100 or more channels. The number of time points required for training may be as few as several times the number of unmixing weights (usually the square of the number of channels), though using more (clean) training data are preferable. In turn, the number of channels must be at least equal to (and preferably larger than) the number of interpretable components to be separated (Makeig et al. 1999a).

The success of ICA applied to EEG data is strictly determined by the degree to which EEG dynamics fit the ICA model. The first requirement, that the underlying sources mix linearly in the electrode recordings, is assured by the biophysics of volume conduction at EEG frequencies (Nunez 1981). The assumption of relative spatial stationarity of EEG sources is compatible, at least, with evidence of brain modularity from anatomy and functional imaging. The assumption of relative independence of the source signals is compatible with physiological models that emphasize local, short-range intracortical and radial thalamocortical coupling in the generation of local electrical synchronies in the EEG range (Salinas and Sejnowski 2001).

The ultimate validity of the assumptions above in any data set cannot be guaranteed a priori. The consistency and physiologic plausibility of the results of ICA decompositions, such as we present here, including their often tight linkage to behavioral and cognitive variables, are strong indirect evidence for the workability of the assumptions and of the ICA model. Direct physiological testing of the model and its physiologic assumptions will require development of multiscale recording methods. Meanwhile, yet more flexible (but also more intricate) ICA models of EEG activity are possible (e.g., Anemueller et al. 2003). It remains to be seen, however, whether the information gain offered by such models exceeds the loss of statistical power associated with their higher complexity.

As first demonstrated by simulations (Makeig et al. 2000), when training data consist of fewer large source components than channels, plus many more small source components, as might be expected in actual EEG data, large source components are accurately separated into separate output components, with the remaining output components consisting of mixtures of smaller source components. In this sense, performance of the infomax ICA algorithm degrades gracefully as the amount of noise in the data increases. For more details about applying ICA to ERP and EEG data, see Makeig et al. (1999a, 2002) and Jung et al. (2000a, 2000b).

Here, the runica algorithm (available for download with the EEGLAB toolbox of Delorme and Makeig [2004a] from http://sccn.ucsd.edu/eeglab) was applied to sets of 400 to 600 1-s trials (31 channels, 256 time points) time-locked from −200 ms before to +800 ms after onsets of target stimuli presented at any of the five stimulus locations (see Figure 1). Target trials in which the subject did not respond with a button press (fewer than 5%) were removed from the data. Learning batch size was 50. Initial learning rate began near 0.0004 and gradually reduced to 10^−6^ during 50–150 training iterations that required about 30 min of computer time. Results of the analysis were relatively insensitive to the exact choice of learning rate or batch size. Reducing the stopping weight change from 10^−6^ to 10^−7^ did not appear to change the resulting decompositions qualitatively, although when decomposing data from many more channels, we have since noted an advantage to continuing to train until weight change falls below 10^−7^.

#### Component clustering

Commonly in ERP research, neural activity expressed in periocular data channels is ignored for fear of mislabeling eye-activity artifacts as brain activity. Some ICA components of EEG records can be clearly identified as accounting primarily for eye movements, line or muscle noise, or other artifacts through their characteristic scalp maps and activity time courses (Makeig et al. 1996; Jung et al. 2000a, 2000b). Subtracting the projections of artifactual components from averaged or single-trial data can eliminate or strongly reduce these artifacts while preserving the remaining nonartifactual EEG phenomena in all of the data channels. ICA thus makes it possible to examine periocular EEG activity apart from eye movements.

Here, the total of 465 (31 times 15) component maps and mean activity log spectra from the 15 subjects were clustered by applying a modified Mahalanobis distance measure (Enghoff, 1999 see Appendix of Jung et al. 2001b) to vectors coding differences in the component 31-channel (x, y) map gradients and activity log spectra after reduction to 12 and five dimensions respectively by principal component analysis. Cluster membership was in a few cases then further adjusted by eye for uniformity. Clustering based on scalp map gradients and activity spectra, as reported here, is one of several possible component clustering approaches, whose relative advantages have not yet been explored.

#### Event-related spectral dynamics

To examine stimulus- and response-induced changes in the EEG spectrum, we computed ERSP transforms (Makeig 1993) for each channel and each clustered independent data component using the publicly available EEGLAB toolbox (Delorme and Makeig 2004a, 2004b). ERSPs show changes in decibels from baseline in spectral power across a broad frequency range (here, 3–50 Hz). The time/frequency analysis used Hanning-windowed sinusoidal wavelets of 3 cycles at 3 Hz, rising linearly to about 15 cycles at 30 Hz. This modified wavelet transform was selected to optimize the trade-off between temporal resolution at lower frequencies and stability at higher frequencies.

Constructing surrogate data sets by shuffling the data epoch subwindows used to construct the time-locked spectral average allowed choosing an initial within-subject significance cutoff (not corrected for multiple comparisons) of *p* < 0.01. To construct between-subject mean ERSPs, we used binomial statistics to select a significance cutoff based on the minimum number of subjects required to have significant differences (in the same direction) from baseline at a given time/frequency point (*p* < 0.0001). ERSP transforms of the data were computed at each channel and then for each clustered data component. To test for partial phase locking (i.e., nonrandom phase relationships) between EEG processes and the occurrence of experimental events across trials, we used intertrial phase coherence (Makeig et al. 2002).

To test the presence of nonrandom phase relationships (possibly including fixed delays) between activities in different (maximally) independent components, we performed event-related phase coherence analysis (Makeig et al. 2002; Delorme et al. 2002), again with a single-subject bootstrap significance threshold of *p* < 0.01 (uncorrected) between pairs of independent components (from the same subject) included in each pair of independent component clusters (defined below). To exclude the possibility that the observed phase linkages arose only from common phase locking of the portion of the single-trial data constituting the ERP, we also subtracted the concurrent mean ERP from each trial before computing phase coherence. Functions to compute and plot the time/frequency measures used here are also available in the EEGLAB toolbox.

#### Equivalent dipole modeling

Both simple anatomic considerations and observed results of ICA decomposition suggest that cortical EEG sources may be usefully modeled as patches of cortex with partially synchronous local field activities. In brief, the high local coupling density of both excitatory and, particularly, inhibitory neurons in cortex means that local field potentials sufficiently synchronous to create measurable EEG signals should tend to extend through a compact spatial domain—roughly speaking, a patch of cortex of unspecified extent. Through volume conduction, partially synchronous activities within cortical source patches produce far-field potentials throughout the brain and on the scalp. The distribution of the scalp electrical field produced by such a source patch is nearly identical to that of a small dipolar potential element whose geometry is like a tiny flashlight battery oriented perpendicular to the cortical surface. This “battery” is termed the equivalent dipole for the cortical source.

Here, we used a relatively simple and well-known method for fitting the positions and orientations of equivalent dipoles in a four-shell spherical head model for each component (BESA; Megis Software, Munich, Germany). To reduce the time required to process the 485 component maps, we used a version (3.0) of the BESA software that allowed batch processing to fit single-dipole models to each component scalp map. (A dipfit tool set by R. Oostenveld, producing equivalent results, is now available at http://sccn.ucsd.edu/eeglab/dipfit.html). Some bilaterally symmetric component maps were better fit with symmetric dual-dipole models. The successful fits of single-dipole models for many of the clustered components is compatible with their generation within a single, compact patch of cortex, while bilateral dual-dipole models are compatible with tightly coupled oscillatory activity (without net phase delay) in two bilaterally symmetric cortical patches densely connected via corpus callosum or common subcortical drive.

To distinguish the relative regional locations of the component clusters, the scalp maps for the individual components in each cluster were first oriented similarly (e.g., so as to all be positively correlated), normalized, and averaged. These cluster mean maps were then fit with single-dipole models to roughly illustrate the regional distinctions between the sources of the component clusters. Finally, the cluster-mean dipoles and event-related time/frequency information measured by ERSP, intertrial phase coherence,and event-related phase coherence analysis of all the single trials were visualized together in three dimensions using an animated display developed by Delorme et al. (2002).

**Video 1:** Postmotor Theta Dynamics An animation representing grand mean patterns of event-related dynamics in the theta band. Black traces in the lower panel show the envelope of the grand mean ERP time-locked to the subject button press; the dotted vertical line shows the median time of stimulus onset (response minus 352 ms). Theta dynamics computed in a window (center frequency 4.87 Hz, 3-cycle Hanning taper) centered (blue vertical line) 89 ms after the button press (red vertical line). Each sphere in the upper panel represents the location of the equivalent dipole for a component cluster. Approximate projections of the equivalent-dipole locations are shown in shadow on three planes from an average magnetic resonance image (Montreal Neurological Institute). Log spectral power changes (relative to prestimulus baseline) are indicated by the sizes of the spheres (see key, bottom right). Nongrey sphere colors indicate consistent intertrial phase locking (intertrial phase coherence). Colored cylinders joining spheres indicate significant event-related phase coherence between cluster components. (4.26 MB MOV)

## Abbreviations

CM: central midline
EEG: electroencephalographic
ERP: event-related potential
ERSP: event-related spectral perturbation
FM: frontal midline
ICA: independent component analysis
RT: reaction time
